# HepFREEPak: protocol for a multi-centre, prospective observational study examining efficacy and impact of current therapies for the treatment of hepatitis C in Pakistan and reporting resistance to antiviral drugs: study protocol

**DOI:** 10.1186/s12889-023-17290-3

**Published:** 2023-12-18

**Authors:** Ambreen Arif, Aliya Hasnain, Auj Chaudhry, Muhammad Asim, Muhammad Nabeel Shafqat, Abeer Altaf, Noor Saba, Polychronis Kemos, M. Azim Ansari, Eleanor Barnes, Chris Metcalfe, Peter Vickerman, Huma Qureshi, Saeed Hamid, Asad Ali Choudhry, Saad Khalid Niaz, Graham R. Foster, Naheed Choudhry

**Affiliations:** 1 Doctor’s Plaza, Khayaban E Iqbal Block 9 DO Talwar, Karachi, Clifton 75600 Pakistan; 2 Department of Medicine, Aga Khan University, Stadium Road, Karachi, 74800 Pakistan; 3 Gut & Liver Center, Chaudhry Hospital and PARSA Trust Liver Clinic, Gujranwala, Pakistan; 4 Dow University of Health Sciences, Karachi, Pakistan; 5 Centre for Immunobiology, Blizard Institute, Barts and The London School of Medicine and Dentistry, Queen Mary University of London, London, UK; 6 Nuffield Department of Medicine, University of Oxford, Oxford, UK; 7 Oxford University Hospitals NHS Foundation Trust, Oxford, UK; 8 Population Health Sciences, Bristol Medical School, University of Bristol, Bristol, UK

**Keywords:** HCV; Hepatitis C, Screening, Prospective observational study, Multi-centre cohort, Pakistan

## Abstract

**Background:**

Pakistan has one of the highest burdens of Hepatitis C virus (HCV) infection globally. To achieve the World Health Organization’s goals for HCV elimination, there is a need for substantial scale-up in testing, treatment, and a reduction in new infections. Data on the population impact of scaling up treatment is not available in Pakistan, nor is there reliable data on the incidence of infection/reinfection. This project will fill this gap by providing important empirical data on the incidence of infection (primary and reinfection) in Pakistan. Then, by using this data in epidemic models, the study will determine whether response rates achieved with affordable therapies (sofosbuvir plus daclatasvir) will be sufficient to eliminate HCV in Pakistan.

**Methods:**

This prospective multi-centre cohort study will screen 25,000 individuals for HCV antibody (Ab) and RNA (if Ab-positive) at various centers in Pakistan- Karachi (Sindh) and Punjab, providing estimates of the disease prevalence. HCV positive patients will be treated with sofosbuvir and daclatasvir for 12-weeks, (extended to 24-weeks in those with cirrhosis) and the proportion responding to this first-line treatment estimated. Patients who test HCV Ab negative will be recalled 12 months later to test for new HCV infections, providing estimates of the incidence rate. Patients diagnosed with HCV (~ 4,000) will be treated and tested for Sustained Virological Response (SVR). Questionnaires to assess risk factors, productivity, health care usage and quality of life will be completed at both the initial screening and at 12-month follow-up, allowing mathematical modelling and economic analysis to assess the current treatment strategies. Viral resistance will be analysed and patients who have successfully completed treatment will be retested 12 months later to estimate the rate of re-infection.

**Conclusion:**

The HepFREEPak study will provide evidence on the efficacy of available and widely used treatment options in Pakistan. It will also provide data on the incidence rate of primary infections and re-infections. Data on incidence risk factors will allow us to model and incorporate heterogeneity of risk and how that affects screening and treatment strategies. These data will identify any gaps in current test-and-treat programs to achieve HCV elimination in Pakistan.

**Study registration:**

This study was registered on clinicaltrials.gov (NCT04943588) on June 29, 2021.

**Supplementary Information:**

The online version contains supplementary material available at 10.1186/s12889-023-17290-3.

## Introduction

Globally an estimated number of 58 million people have chronic hepatitis C infection, with about 1.5 million new infections occurring yearly [1]. Hepatitis C virus (HCV) is endemic in Pakistan. A systematic review of data from 2010–2015 showed that HCV sero-prevalence among the general adult Pakistani population was 7.5%, with active infection found in 4% of the population [2, 3]. Eliminating HCV in Pakistan requires large-scale screening and reflex testing for HCV ribonucleic acid (RNA) followed by treating [4] those found positive. Currently, the epidemic in Pakistan is expanding, and there is a lack of empirical data on the incidence of infection, reinfection and evidence demonstrating the impact of scaling up treatment [5]. Multiple strains contribute to the ongoing transmission in Pakistan, including genotype 3b (G3b), which is resistant to the current NS5A inhibitors and poses a challenge to treatment with sofosbuvir-based regimens. Virological failure rates with the available treatment (sofosbuvir and daclatasvir; SOF/DAC) in Pakistan are under-reported, and resistance in those who do not respond is unexplored [6–9]. If the population who fail treatment, develop resistance and are not retreated effectively, it is likely to leave an expanding, drug-resistant population. In the long run these drug-resistant viruses might transmit leading to reduced efficacy of currently available first-line regimens.

In Pakistan, the most effective HCV treatments for genotype 3 are unavailable and unaffordable (glecaprevir/pibrentasvir – sofosbuvir/velpatasvir). The available and affordable treatment with sofosbuvir and daclatasvir in adjacent India [10] cured 85.2% of patients in an intention-to-treat analysis. This study did not identify a reduced response in patients with cirrhosis, but another similar study from the USA using SOF/DAC in genotype 3 patients showed lower cure rates (Sustained Virological Response; SVR)—approximately < 70% in those with cirrhosis [11]. However, local studies in Pakistan demonstrate a high response rate of > 95% [12–14] with SOF/DAC, yet there is still uncertainty around current treatment regimes. Daclatasvir, targets a viral component that is polymorphic and common mutations reduce treatment efficacy. For the more potent NS5A inhibitor velpatasvir, NS5A polymorphisms in people with genotype 3 may reduce response in those with cirrhosis by 12% [15] and a similar impact with daclatasvir might be anticipated. Additionally, we have found that in patients with genotype 3 HCV and cirrhosis, a common polymorphism, V at position 150 in the HCV genotype 3a NS5b polymerase, combined with a rare combination of variants in genotype 3, reduced the response to sofosbuvir by several fold [16] thus potentially, decreasing the effectiveness of treatments used in Pakistan.

### Study objectives

The primary objectives of the HepFREEPak observational and modelling study are to determine: (i) the effectiveness of current HCV therapy in Pakistan with SOF/DAC by determining the sustained virological response rate (SVR); (ii) the incidence of new infections after 12-months in people who initially tested HCV antibody negative; (iii) the incidence of reinfection after an interval of 12–18 months in those who achieved an SVR after completing treatment with DAAs;(iv) emergence of viral resistance-associated variants in people who do not respond to first-line DAAs and, (v) model the impact and cost-effectiveness of scaling up testing and treatment in Pakistan, including retreatment of those failing treatment.

The secondary objectives are to determine (i) health-related quality of life in people with chronic HCV infection compared to those who are uninfected, (ii) healthcare costs associated with HCV infection; (iii) costs of testing and treating people for HCV infection in Pakistan; (iv) risk factors for all primary and new HCV infections as well as HCV reinfections compared to those who have tested negative; (v) viral polymorphisms in HCV that are associated with a non-responsiveness to antiviral therapy.

## Methods

HepFREEPak is a prospective multi-centre, observational study. The study is funded by the Wellcome Trust (220866/Z/20/Z) and sponsored by Queen Mary University of London (QMUL). This study was registered on clinicaltrials.gov (NCT04943588) on June 29, 2021.

### Study design & setting

**Fig. 1:**
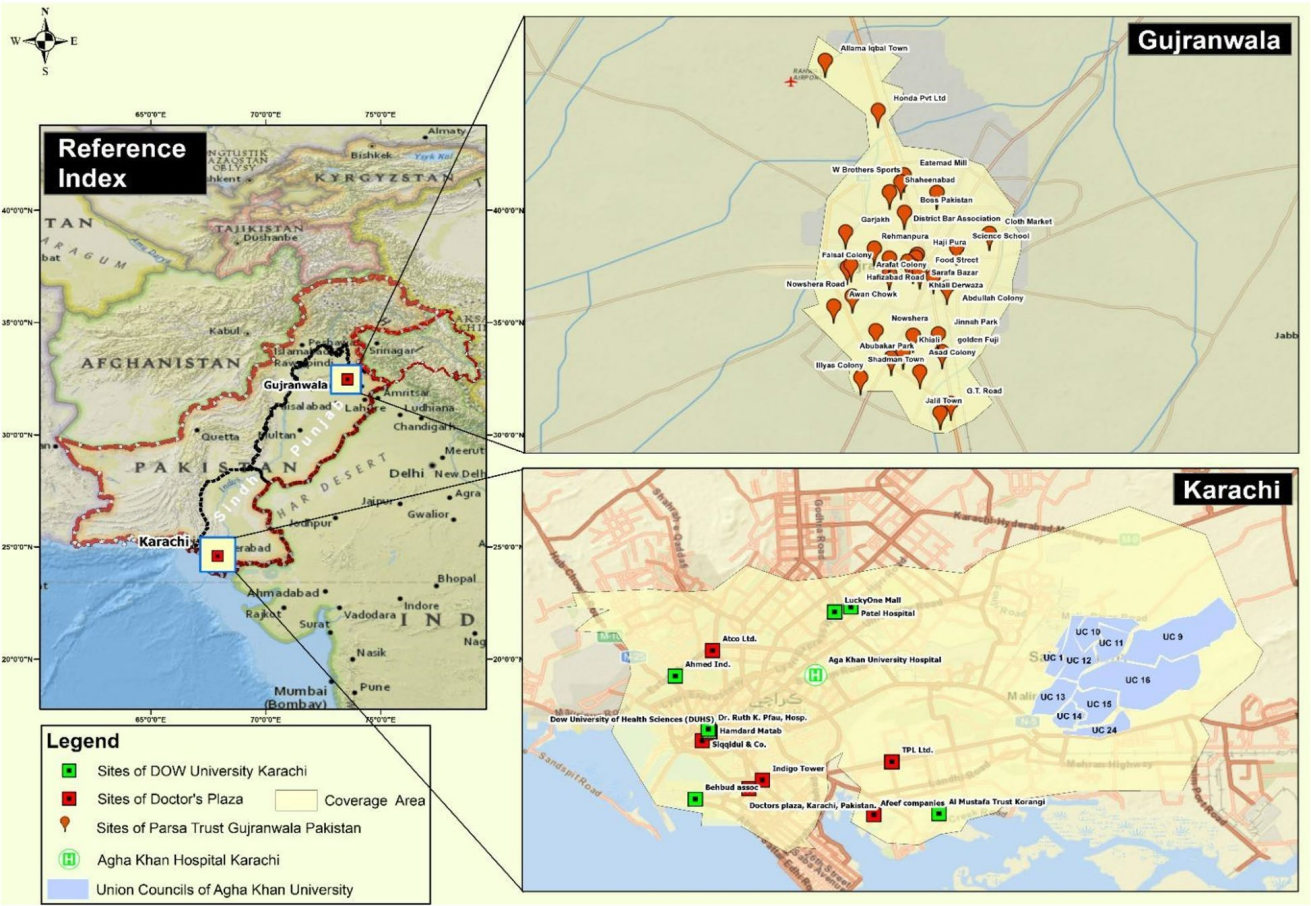
Map showing the main HCV screening study sites in the cities of Karachi and Gujranwala, Pakistan along with their catchment areas for recruitment. The specific icons demonstrate the localized pop-up clinics/catchment areas covered by each site. This map was adapted from Google Maps. https://www.google.com/maps/d/viewer?hl=en&mid=1A5pcd3_Wi5CM-JCjgFwW6w8Gvz47lfk&ll=28.572149991669296%2C70.52872740000001&z=6

The study started enrollment on November 1st, 2021, and is expected to conclude by December 31st, 2024. The study is being carried out at four sites across two cities in Pakistan, where eligible participants are enrolled in various study groups described in Additional file 1: Appendix 1. Figure 1 shows the four study sites selected for this study with three sites in Karachi, Sindh (Aga Khan University, Dow University of Health Sciences, the Liver Clinic at Doctors’ Plaza) and the fourth site in Gujranwala Lower Punjab (Gut & Liver Center and PARSA Trust Liver Clinic).

### Study eligibility

This study will enroll 25,000 participants based on the eligibility criteria described in Table 1. Suitable participants are recruited via different strategies to maximize diversity in the population tested. Local pop-up clinics (also known as screening camps) in Gujranwala are organized by local politicians (known as Nazims) and doctors in locally accessible spaces. The testing is advertised to the community through mosques, word-of-mouth, banners and leaflets. Screening of neighbourhoods in Malir (Karachi) consists of approaching residents, door-to-door and offering the HCV test at home by clinical staff. Other participants enrolled in the study in Karachi are via community advertising of the screening clinics at hospital receptions, when patients and relatives are attending for other medical issues, or through local non-governmental organisations that encourage companies with large labor workforces to allow pop-up clinics within the company premises to test staff over a few days as part of their wellbeing policies.

**Table 1:** Study inclusion & exclusion criteria

Inclusion criteria	Exclusion criteria
Adults over 18 years of age Willing to undergo hepatitis C testing Able and willing to give informed consent Willing to return in 12 months’ time for repeat testing Resident in the area and not planning to leave the region	Unwilling to give consent Unwilling or unable to undergo the necessary procedures Clinically significant illness (other than HCV) or other major medical condition that may interfere with the subject's treatment, assessment, or compliance with the protocol Comorbidities limiting life expectancy to less than 12 months

### Study processes and intervention

**Fig. 2:**
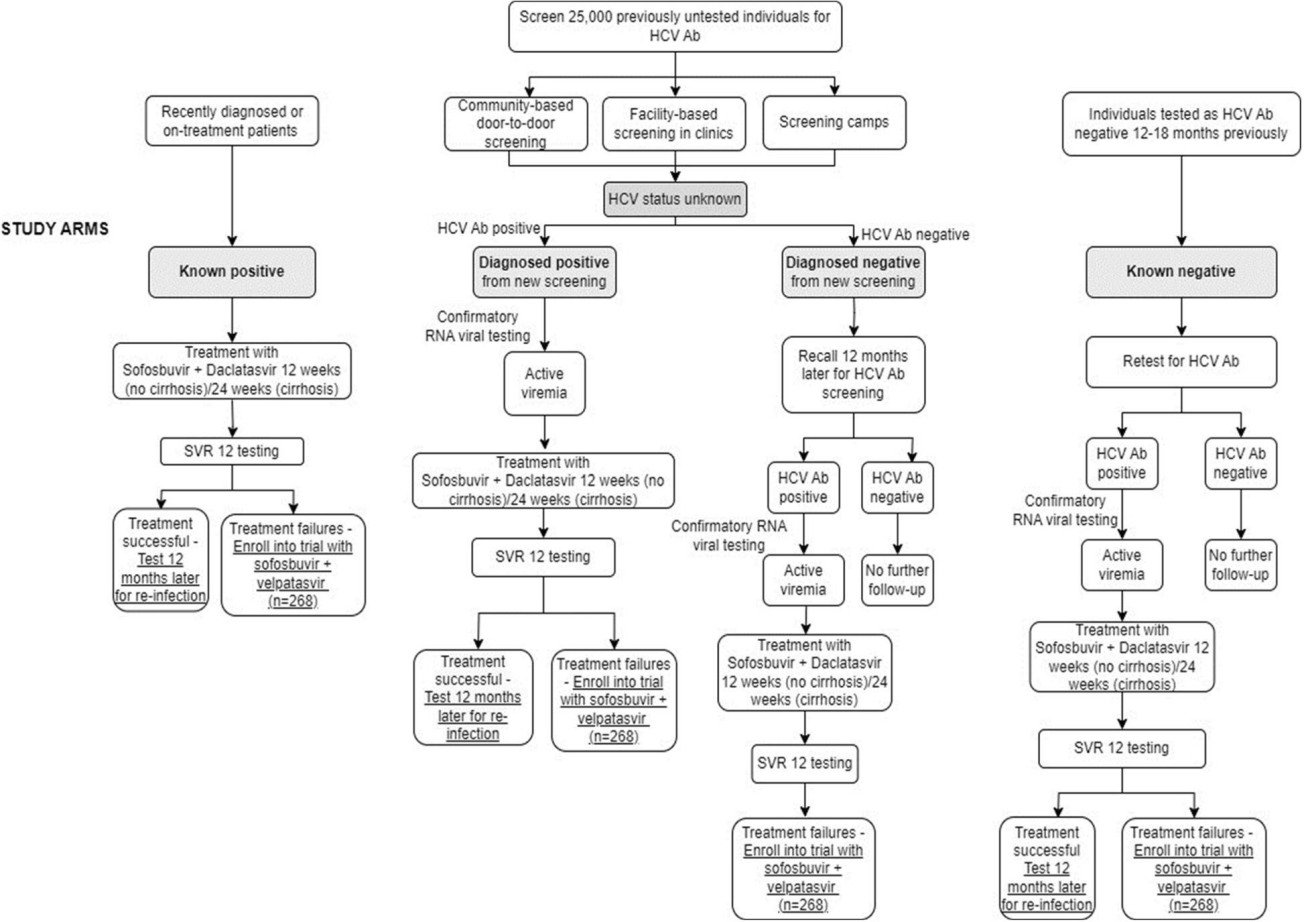
Study Flow Diagram. The four separate study groups of participants (see Additional file 1: Appendix 1 for full details). The known HCV-positive subjects that have not started treatment have a confirmatory RNA test before treatment commences and then are tested for SVR12 weeks after the completion of the SOF/DAC treatment regime, to assess first-line treatment response. After 12-months, these subjects are re-tested for HCV RNA to establish re-infection rates. HCV Ab-positive subjects at the screening stage (diagnosed HCV positives), follow a similar study pathway to assess first-line treatment response and then assess re-infection rates after 12-months. HCV Ab negative participants at screening (diagnosed HCV negatives) are re-tested 12 months later to establish HCV incidence rates. Finally, known HCV-negative subjects have previously been tested 12–18 months prior to recruitment and are tested to assess HCV incidence

The study is outlined in Fig. 2. Enrolled participants will be screened initially using a rapid finger prick test (WHO pre-qualified-SD Bioline /Intec). Individuals with a positive screening result will undergo reflex phlebotomy, where 10 ml venous blood is drawn for HCV RNA testing along with complete blood count (CBC), aspartate aminotransferase (AST), alanine aminotransferase (ALT), serum creatinine and hepatitis B surface antigen (HBsAg). Active viremia will be confirmed by detecting HCV RNA using GeneXpert (Cepheid, France) or HCV core Antigen (HCV cAg) on the ARCHITECT platform (Abbott Diagnostics, Wiesbaden, Germany). Plasma samples of individuals with active viremia will be archived before treatment.

### Treatment regimens

AST to Platelet Ratio Index (APRI) with or without the Fibrosis-4 (FIB-4) Index for Liver Fibrosis index will be used to determine the presence of cirrhosis. Patients with no evidence of cirrhosis, (APRI cut-off of < 1.5 and no contraindication to treatment) will be prescribed SOF/DAC for 12 weeks after evaluation by a specialist physician. Patients identified as having cirrhosis (APRI ≥ 1.5) without signs and symptoms of decompensation are to be prescribed SOF/DAC for 24 weeks. Patients who do not respond to first-line treatment with SOF/DAC based on their SVR result (12-weeks post completion of treatment; SVR12) will be offered enrollment in a linked randomised controlled trial of second line treatment with sofosbuvir and velpatasvir.

### Statistical analysis

#### Sample size and power calculation

Around 25,000 participants will be recruited. Incidence in the high prevalence settings (10% or over) where we will be screening is likely to be around 1.0 per 100 person-years (/100pyrs) based on our experience and estimates. Our aim is to detect incidence rates with sufficient precision to allow robust modelling and to detect (through questionnaires) major risk factors associated with heightened infection risk, so justifying significant investment to reduce their impact. Potential risk factors include greater use of medical injections or medical procedures, injection drug use, use of barbers or dentists, and possibly childbirth.

For the primary incidence study, 20,000 HCV antigen-negative participants with 80% follow-up (16,000) would estimate a true incidence of 1.0/100pyrs with 95% confidence intervals 0.85 to 1.17/100pyrs. Assuming 25% are exposed to a significant risk factor, 16,000 participants give 92% power at 5% significance to detect a true increase in risk from 0.83/100pyrs in unexposed to 1.5/100pyrs in the exposed (Rate Ratio = 1.81). Given that these are uninfected individuals and therefore may be at lower risk, consider a risk factor to which 10% are exposed, 16,000 participants would give 84% power at 5% significance to detect an increase in risk from 0.91 to 1.8/100pyrs in the exposed (Rate Ratio = 1.98).

For the reinfection incidence study, we need 5,000 treated patients to generate sufficient subjects for the linked treatment failure trial. Given 4,000 ‘cured’ participants in follow-up, we would estimate a true incidence of 1.0/100pyrs with 95% confidence interval 0.72 to 1.36/100pyrs. Given prior infection these individuals are likely to have been exposed to a significant risk factor in the past. Assuming 25% are exposed to a significant risk factor, 4,000 participants give 82% power at 5% significance to detect an increase in risk from 0.70/100pyrs in unexposed to 1.9/100pyrs in the exposed (Rate Ratio = 2.71). Hence, we will be able to detect strong, ongoing risk factors for re-infection that justify interventions in those receiving treatment.

Rate ratios will be estimated, in Poisson regression models, as the coefficient for an indicator variable that distinguishes individuals exposed and unexposed to a risk factor. Rate ratios will be presented with 95% confidence intervals and *p*-values. Estimated rates, risks and prevalences will be presented with 95% confidence intervals. Estimate incidence will be adjusted for sex assigned at birth (male/female), difference in ages and the screening settings.

### Data collection and assessments

The study team will manually record all participants’ data on a paper-based case report form (CRF) in English or translated into Urdu (validated for use). The data (including socio-demographic- see Additional file 1: Appendix 2, and questionnaires) will be entered into a custom-built Research Electronic Data Capture (REDCap) database hosted at QMUL. REDCap is a secure, web-based software platform designed to support data capture for research studies [17, 18].

#### Consent to participate

At enrollment, eligible participants will be asked to give their informed consent confirmed by an independent witness. A pre-treatment questionnaire will be filled out to record demographics and answer risk factors, quality of life (QOL), productivity and healthcare usage questions (Table 2). Participants will complete the questionnaires after blood sampling, but before they are aware of their HCV status. This will ensure that quality of life data reflects health status without knowledge of infected status. We will sample 1200 people (200 HCV positives and at least 1000 HCV negatives; 1:5 ratio) – see power calculation above.

**Table 2:**
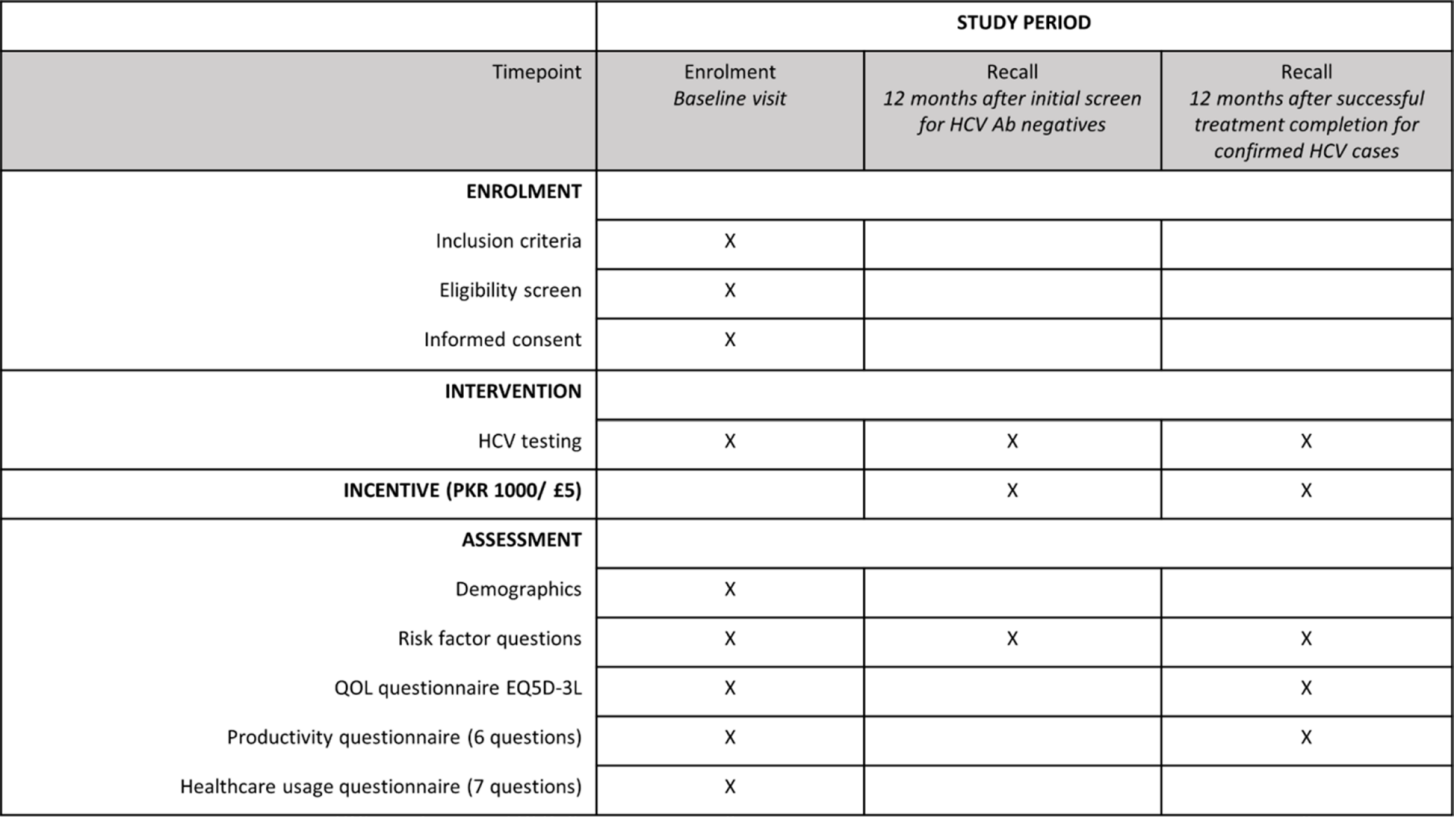
Study Timelines including baseline visit, 12 months recall for HCV Ab negative individuals and HCV positive patients after successful treatment

*Abbreviations: QOL* Quality of life, *EQ5D-3L* Generic tool for patient-reported outcomes like quality of life [19]

HCV Ab negative individuals will be recalled for repeat HCV Ab testing after 12 months from screening (range 12–18 months). They will also be asked to complete risk factor questions at this time (Table 2). Those who were HCV RNA positive at screening and undergo first-line treatment will be retested for viraemia 12 weeks after completion of treatment to assess SVR. Those that achieve SVR will be recalled for further testing 12 months (range 12–18 months) after the completion of the HCV treatment regimen and will be asked to complete questionnaires on risk factors, QOL EQ5D-3L and productivity again (Table 2).

### Risk factors

Before receiving their screening HCV results, a risk factor questionnaire will be completed by all those testing HCV positive and the next 5 subjects found to be HCV negative at that testing site. HCV-positive participants and matched controls during the 12-month follow-up periods will be reassessed on these questions (part of incidence study).

### Health-related Quality of Life (HRQOL) and productivity

We will assess health-related quality of life in a subset of people screened for HCV infection. They will be asked to complete an EQ-5D-3L quality of life and productivity questionnaire before they are aware of their HCV status. This will ensure that quality of life data reflects health status without knowledge about their infected status.

### Health usage

A survey instrument to estimate the healthcare standard of care costs related to HCV infection in the last year will be performed at the pre-screening stage, at the same time as when they complete the EQ-5D-3L and productivity questionnaire. This will ask about access to inpatient and outpatient health facilities in the last year and estimate associated out-of-pocket costs.

### Study and data monitoring

Oversight for this study is provided by:

A study management group comprising the PIs, collaborators, grant holders, statisticians, and the study coordination teams.

A steering committee to monitor and supervise the progress of the study towards its interim and overall objectives and review at regular intervals (annual/bi-annual/quarterly) relevant information from other sources (e.g., other related studies).

Data monitoring will be performed from the inception of participant enrolment at each site. The investigator will ensure that all data on the CRFs are complete, accurate, and consistent with source documentation. The investigator and study centre will permit study-related monitoring, audits, Institutional review boards (IRB)/independent ethics committee (IEC) review and regulatory inspection by providing authorised personnel from the sponsor, its representatives, the IRB, and appropriate regulatory agency direct access to all study-related data. A clinical monitor (or designee) will review the CRFs for completeness and accuracy. Data entered on REDCap will be verified against information on original source documents for completeness, accuracy, and plausibility. The study sponsor at QMUL and the PIs retain the right to audit study sites.

### Research ethics approval, consent, and confidentiality

The study is being conducted in accordance with the UK Policy Framework for Health and Social Care Research.

This study was reviewed and approved by the Queen Mary Ethics Review Committee (Reference number QMERC20.306) on 27th May 2021, the Institutional Review Board of Aga Khan University (Reference number: 2021–6341-18,329) on 29th June 2021 and Dow University of Health Sciences (Approval number: 2139/DUHS/Approval/2021/463) on June 12th 2021. The study was approved by the National Bioethics Committee (NBC) Pakistan (Ref: No.4–87/NBC-677/21/439) on 9th Sep 2021.

There is negligible risk to the study participants from the study medication (which has already been assessed for safety and is widely used [20]. Written informed consent will be obtained from all eligible participants after explaining details of the study and available treatment options.

All the information related to the participants, CRFs, study data, and all communications will be kept secure. Access to the information is to be provided only to the study team members. All the data in hardcopy will be kept in locked cabinets. Electronic data will be password protected. Data will be shared for study-related monitoring, audits, IRB/IEC review, regulatory inspections, and sponsor or the sponsor designee. To maintain patient privacy, all CRFs, study drug accountability records, study reports, and communications will identify the patient by the assigned patient number. The Investigator will grant monitor(s) and auditor(s) from the Sponsor or its designee and regulatory authority(ies) access to the patient’s original medical records for verification of data gathered on the CRFs and to audit the data collection process.

## Discussion

Globally, 78.6% of individuals with chronic hepatitis C remain undiagnosed [21] and active case finding for undiagnosed individuals within the community is a pivotal determinant for effective HCV elimination. However, it is not yet clear whether active case-finding and therapy with sub-optimal, but affordable, regimens will be sufficient to reduce infection rates to a level at which viral elimination can be achieved. Given the increasing pressure on scarce health care resources, it will be important to determine the probable health care gains from any community case-finding program before large-scale implementation of expensive elimination programs.

This paper describes the protocol for a prospective multi-centre observational study, actively recruiting in Pakistan. The study protocol integrates the clinic facility, community and corporate sector multi-centre models for adaptive screening, diagnosis, and timely HCV treatment. The study protocol includes an estimation of EQ-5D-3L, health care costs, productivity assessment and risk factors for modelling and economic evaluation allowing policy advocacy around cost-effective interventions and strategies for HCV elimination.

The current HCV screening and treatment strategies in Pakistan are insufficient, and ongoing HCV micro-elimination efforts may fall short of achieving the WHO 2030 elimination targets [22]. To persuade healthcare leaders to undertake an expansion of the current program will require evidence-based support for the benefits of the program and this unique multi-centre study will provide the data needed to support or refine an elimination program in Pakistan and will likely serve as a paradigm for studies elsewhere. We anticipate that the outputs from the study will be a recommendation to either continue with the current strategy, with a modified elimination strategy (perhaps using more expensive treatment options for a subset of ‘difficult to cure’ patients) or to abandon the program as futile and divert resources to other health care initiatives.

### Strengths

This study, when complete, will be the first of its kind in Pakistan with a large well-representative sample size to assess the incidence of HCV in individuals from multiple centers through multiple approaches. Recruitment, interviews, rapid on-spot testing and treatment at multi-centers and community sites will allow one-time testing and linkage to care, thus avoiding delays. This study will also report the incidence of virological failure rates and reinfection in a large, real-world setting following the introduction of DAA therapies in Pakistan.

This study will also provide evidence to policymakers on how an individual’s HCV status impacts health-related quality of life and productivity and if patients with advanced HCV infection have higher associated health costs thus allowing comparisons of healthcare gains in this program to be compared to other health care initiatives.

### Limitations

The study has some limitations. The study is centered on two main cities in Pakistan which may reduce our ability to draw more general conclusions. However, we have chosen diverse settings within the testing and treatment sites, and we believe that our findings are likely to be sufficiently robust to allow general conclusions to be drawn. Our major limitation is likely to be the proportion of people who attend for repeat testing and there is a danger that those who attend may be atypical. Our power calculations are conservative, and we anticipate that, with incentives, > 70% of patients will be contactable. Given the large sample size and detailed demographics, we believe that we are likely to be able to derive inferences that are applicable to a nationwide program.

In conclusion, based on the study outcomes, we believe that the current study will guide evidence to design an evidence-based framework to eliminate HCV infection in Pakistan.

### Supplementary Information

**Additional file 1: Appendix 1.** Study groups & interventions. **Appendix 2.** Study instruments - Sociodemographic data.

**Additional file 2. **Supplementary material.

## Data Availability

The datasets generated and/or analyzed during the current study are not publicly available due to the reason that the study is still ongoing, but are available from the corresponding author on reasonable request once the study is completed and analyzed.
