# Supplementary material for: HepFREEPak: protocol for a multi-centre, prospective observational study examining efficacy and impact of current therapies for the treatment of hepatitis C in Pakistan and reporting resistance to antiviral drugs: study protocol
Source: BMC Public Health. 2023 Dec 18;23:2529. doi: 10.1186/s12889-023-17290-3 (PMC10726502; doi:10.1186/s12889-023-17290-3)
Supplement: Supplementary file 1 — Additional file 1: Appendix 1. Study groups & interventions. Appendix 2. Study instruments - Sociodemographic data. [file 12889_2023_17290_MOESM1_ESM.docx]

**Appendix**

**Appendix 1.** Study Groups & Interventions

| Enrolment Group/Arm | Definition | Intervention |
| --- | --- | --- |
| 1. Diagnosed Negatives | Individuals with a negative HCV ab test on initial screening | Recall after 12 months for HCV testing to look for new infection |
| 2. Diagnosed Positives | Individuals with a positive HCV ab test on initial screening followed by a detectable viral load on confirmatory testing. | 1. Assessment for cirrhosis using APRI +/- fibroscan 2. Treatment with DAA: Sofosbuvir + Daclatasvir 12 weeks in non-cirrhoticsORSofosbuvir + Daclatasvir for 24 weeks if evidence of cirrhosis 3. Test SVR (11-18 weeks post-treatment)   If treatment successful: Recall after 12 months for HCV testing to look for re-infection (12-18 months)  If treatment failure:  Enrolment in treatment trial with Sofosbuvir + Velpatasvir |
| 3. Known Positives | Individuals who, upon enrolment, or have positive anti-HCV and PCR results, or are on first-line treatment, are about to start treatment or have just completed treatment and are due for SVR testing | 1. Complete treatment using DAAs 2. Test SVR (11-18 weeks post-treatment) 3. Recall after 12 months for HCV testing to look for re-infection (12-18 months) |
| 4. Known negatives | Individuals tested from other studies/programs with a negative HCV Ab result (12-18 months) are being invited to undergo repeat testing looking for new infections. | 1. HCV RNA testing if they have a positive screening result followed by treatment with DAA if treatment eligible.   If an HCV positive result is detected at this stage, they are categorized as ‘New Positives from Known HCV Negative’. |

**Appendix 2.**

*Study Instruments - Sociodemographic data*

Data on personal identifiers (Name, date of birth, phone number and National ID card number),as well as personal demographics – gender, living situation, ethnicity, marital status, education level and occupation, will be collected. The data will be anonymised on REDCap. The personal identifiers will be used by the site teams to contact recruited subjects for 12-month follow-ups.

**Additional file Figure 1:** This map illustrates the 4 main HCV screening study sites in cities of Karachi and Gujranwala, Pakistan along with their catchment areas for recruitment. The specific icons demonstrate the camp sites/catchment areas covered by each site. This map was adapted from google maps

**Figure 2**: Study Flow Diagram including Screening Trial (community-based door-to-door screening, facility-based in-clinic, screening camps) Linkage to care (assessment, treatment and follow-up). Abbreviations HCV hepatitis C virus, SVR sustained virological response, HCV Ab hepatitis C antibodies, RNA ribonucleic acid.

**Table 1**: Study Inclusionand exclusion criteria

**Table 2**: Study Timelines including baseline visit, 12 months recall for HCV negative individuals and HCV positive patents after successful treatment.
