# Supplementary material for: HepFREEPak: protocol for a multi-centre, prospective observational study examining efficacy and impact of current therapies for the treatment of hepatitis C in Pakistan and reporting resistance to antiviral drugs: study protocol
Source: BMC Public Health. 2023 Dec 18;23:2529. doi: 10.1186/s12889-023-17290-3 (PMC10726502; doi:10.1186/s12889-023-17290-3)
Supplement: Supplementary file 2 — Additional file 2. Supplementary material. [file 12889_2023_17290_MOESM2_ESM.docx]

**SUPPLEMENTARY MATERIAL**

Sentinel Site 1 -Aga Khan University Hospital, Karachi

The Aga Khan University Hospital primarily recruits participants from the Malir district of Karachi, the city’s largest district with a population of approximately 2 million comprising mixed rural and peri-urban settlements. The rural communities of Malir are recognized “hot spots” where HCV prevalence is much higher than the national average of 6%^[[1]](#endnote-2)^. In a systematic population-based seroprevalence survey of HCV in this resource-limited area of Karachi, the HCV antibody positivity was 23.4%^[[2]](#endnote-3)^.

New screening (enrollment into the “HCV status unknown” category/arm) and re-screening for incidence (HCV status known negative) will be performed through a community-based approach in selected union councils (UCs) of the district. Geographical mapping will identify and label households as “structures”. Study staff will sequentially conduct door-to-door visits to identify and recruit eligible participants.

A cohort of 1000 individuals known to have a negative screening result as part of previous testing 12 months earlier (HCV status known negative) will be identified from a dataset of a previous micro elimination program within the same catchment area performed using the same techniques.

As an adjunct to a community-based approach, diagnosed cases of HCV (“known positives”) will also be recruited through remote clinics of Aga Khan University Hospital known as Integrated Medical Services (IMS), which are located across the city.

Decentralizedcascade of care in community setting: Individuals who are found to have a positive screening test on Rapid Diagnostic Test (RDT) have reflex blood drawn for further diagnostic testing. Patients with active viremia are subsequently visited at their homes by a primary care physician for clinical evaluation and prescription of treatment based on treatment eligibility. Free-of-cost treatment (sofosbuvir/daclatasvir) is provided during home visits at monthly intervals to those who have no cirrhosis or have compensated cirrhosis. Those with signs and symptoms of decompensation are referred for specialist care.

Sentinel Site 2: Liver clinic at Doctor’s Plaza, Karachi

The gastroenterology and hepatology clinic at the Doctor’s Plaza in Karachi is a secondary care private clinic with a special focus on liver diseases. The patient population is predominantly from rural lower socio-economic areas of Sindh, who travel to seek specialist care.

New screeningis done in liaison with an NGO-The Health Foundation (<https://thehealthfoundation.org/>), that regularly undertakes HCV screening in the community and the corporate sector. Additionally, independent screening camps are organised in the corporate sector, where the team screens up to 300-400 people daily at the corporation's premises on any assigned day. Screening is by laboratory HCV antibody and RNA testing and positive results are conveyed to the patient by the study team along with an offer of free treatment.

Sentinel Site 3: Liver clinic, Dow University of Health Sciences, Karachi

Dow University Hospital selected six centres across Karachi for outreach screening, some leveraging pre-existing screenings conducted by a charity (The Health Foundation). Most of the recruitment for new HCV screening will beat Patel Hospital, a tertiary care hospital located in a populous urban area of Karachi. The hospital caters to a mixed population from adjacent areas, including some underserved colonies. Patients and their attendants visiting outpatient clinics are to be enrolled in line with the eligibility criteria and screened. Those with confirmed infection will be linked to the specialist hepatology clinic at the hospital for further assessment and management, including HCV treatment.

The remainder of the recruitment will be conducted at screening events at corporate organisations and shopping malls, where diagnosed cases are linked to the specialist clinic at Patel Hospital. The liver clinic at Dow University of Health Sciences is a specialist clinic where patients are referred from all over the city and other cities to seek specialist consultation. Patients with HCV who meet enrolment criteria are enrolled into the “Diagnosed-positive” arm upon treatment initiation.

Sentinel Site 4: Liver clinic, Gujranwala

The Liver clinic located in Gujranwala primarily recruits patients from two main settings,PARSA Trust Liver Clinic andGut & Liver Center, specialised clinics located in tertiary care hospitals. These clinics cater to patients of low socioeconomic status visiting from the local vicinity, which are known to have a high burden of HCV. All patients and their attendants visiting the clinic are offered HCV screening, and those with confirmed infection are followed up in the clinic for further care. Patients visiting the clinic with known HCV infection will also be recruited into the trial.

HCV screening will be conducted at multiple community-based camps, which are organised within the local community and supported by political and religious leaders. The camps are held at a central location in various Union Councils (UC) of the district. To ease the access of the individual enrollment electronic media will be used to encourage the population to attend the camps. PCR tests for those HCV antibody positives with point-of-care testing will be performed through the government’s provincial Hepatitis Control program or through a private trust centre. Those with confirmed infection are then referred to specialist clinics for further care and treatment.

References:

1. Qureshi H, Bile KM, Jooma R, Alam SE, Afridi HU. Prevalence of hepatitis B and C viral infections in Pakistan: findings of a national survey appealing for effective prevention and control measures. East Mediterr Health J. 2010;16 Suppl:S15-23. [↑](#endnote-ref-2)
2. Janjua NZ, Hamza HB, Islam M, Tirmizi SF, Siddiqui A, Jafri W, et al. Health care risk factors among women and personal behaviours among men explain the high prevalence of hepatitis C virus infection in Karachi, Pakistan. J Viral Hepat. 2010;17(5):317-26. [↑](#endnote-ref-3)
